# MR-spectroscopy in metachromatic leukodystrophy: A model free approach and clinical correlation

**DOI:** 10.1016/j.nicl.2022.103296

**Published:** 2022-12-20

**Authors:** Joana Feldmann, Pascal Martin, Benjamin Bender, Lucia Laugwitz, Laimdota Zizmare, Christoph Trautwein, Ingeborg Krägeloh-Mann, Uwe Klose, Samuel Groeschel

**Affiliations:** a Department of Neuropediatrics, Developmental Neurology and Social Pediatrics, University of Tübingen, 72076 Tübingen, Germany; b Department of Neurology and Epileptology, Hertie Institute for Clinical Brain Research, University of Tübingen, 72076 Tübingen, Germany; c Department of Diagnostic and Interventional Neuroradiology, University Hospital Tübingen, 72076 Tübingen, Germany; d Werner Siemens Imaging Center, University of Tübingen, 72076 Tübingen, Germany

**Keywords:** ARSA, Arylsulfatase A, Asp, Aspartate, AUC, Area under the curve, Cho, Choline, Cr, Creatine, CSI, Chemical shift imaging, CST, Corticospinal tract, FWM, Frontal white matter, Gln, Glutamine, Glx, Glutamate/Glutamine, GM, Grey matter, GMFC-MLD, Gross motor function score in metachromatic leukodystrophy, IOI, Interval of interest, IQ, Intelligence quotient, MLD, Metachromatic Leukodystrophy, MRI, Magnetic resonance imaging, MRS, Magnetic resonance spectroscopy, Myo, Myo-inositol, NAA, *N*-acetylaspartate, NMR, Nuclear magnetic resonance, ROI, Region of interest, Metachromatic leukodystrophy, MR spectroscopy, NAA, *N*-acetylaspartate, Urine NMR spectroscopy

## Abstract

**Background and purpose:**

Metachromatic leukodystrophy (MLD) is a lysosomal enzyme deficiency disorder leading to demyelination and subsequently to a progressive decline in cognitive and motor function. It affects mainly white matter where changes during the course of the disease can be visualized on T2-weighted MRI as hyperintense areas. Associated changes in brain metabolism can be quantified by MR spectroscopy (MRS) and may give complementary information as biomarkers for disease characterisation and progression. Our study aimed to further investigate the correlation of MRS with clinical parameters for motor and cognitive function by using a model free MRS analysis approach that would be precise and straightforward to implement.

**Materials and methods:**

53 MRS datasets derived from 29 patients (10 late-infantile, 19 juvenile) and 12 controls were acquired using a semi-LASER CSI sequence covering a slice through the centrum semiovale above the corpus callosum. We defined four regions of interest in the white matter (frontal white matter [FWM] and the cortico-spinal tract [CST] area, each left and right) and one in cortical grey matter. Spectra were analysed using a model and fitting free approach by calculating the definite integral of 10 intervals which were distributed along the whole spectrum. These 10 intervals were orientated towards the main peaks of the metabolites *N*-acetylaspartate (NAA), creatine, myo-inositol, choline, glutamine/glutamate and aspartate to approximately attribute changes in the intervals to corresponding metabolites. Their ratios to the main creatine peak integral were correlated with clinical parameters assessing motor and cognitive abilities. Furthermore, in a post-hoc analysis, NAA levels of a subset of 21 MR datasets were correlated to NAA levels in urine measured by ^1^H (proton) nuclear magnetic resonance (NMR) spectroscopy.

The applied interval integration method was validated in the control cohort against the standard approach, using spectral profile templates of known metabolites (LCModel). Both methods showed good agreement, with coefficients of variance being slightly lower for our approach compared to the related LCModel results. Moreover, the new approach was able to extract information out of the frequency range around the main peaks of aspartate and glutamine where LCModel showed only few usable values for the respective metabolites.

**Results:**

MLD spectra clearly differed from controls. The most pronounced differences were found in white matter (much less in grey matter), with larger values corresponding to main peaks of myo-inositol, choline and aspartate, and smaller values associated with NAA and glutamine. Late-infantile patients had more severe changes compared to later-onset patients, especially in intervals corresponding to NAA, aspartate, myo-inositol, choline and glutamine.

There was a high correlation of several intervals in the corticospinal tract region with motor function (with the most relevant interval corresponding to NAA peak with a correlation coefficient of −0.75; p < 0.001), while cognitive function, by means of IQ, was found to be most correlating in frontal white matter corresponding to the NAA peak (r = 0.84, p < 0.001).

The post-hoc analysis showed that the main NAA peak interval correlated negatively with the NAA in urine (r = −0.584, p < 0.001).

**Conclusion:**

The applied model and fitting free interval integration approach to analyse MRS data of a semi-LASER sequence at 3T suits well to detect and quantify pathological changes in MLD patients through the different courses of the disease and correlates well with clinical symptoms while showing smaller dimensions of variation compared to the more sophisticated single metabolite analysis using LCModel. NAA seems the most clinically meaningful biomarker to use in this context. Its correlation with urine measurements further underlines its potential as a clinically and biologically useful parameter of disease progression in MLD.

## Highlights

• MLD patients show specific metabolic changes throughout the disease course.
• Metabolic changes in different brain regions correlate with clinical symptoms.
• NAA seems the most clinically meaningful biomarker to use in this context.
• NAA in the brain correlates with NAA in urine in MLD-patients.

## 1 Introduction

Metachromatic leukodystrophy (MLD) arises from a lysosomal enzyme arylsulfatase A (ARSA) deficiency and, with a progressive accumulation of sulfatides in cells and myelin sheaths, leads to an increasing severe motor and cognitive disability (Kehrer, 2011, Gieselmann and Krageloh-Mann, 2010, Kehrer, 2021, Fumagalli, 2021). Its prevalence in the northern European and North American populations is estimated with 1:100.000 and is thus one of the most frequent in the group of leukodystrophies (Gieselmann and Krageloh-Mann, 2010, Heim, 1997). A distinction is made between a late-infantile, juvenile and adult form of the disease depending on the age of onset of clinical symptoms. The late-infantile form progresses with a rapid loss of motor and cognitive abilities and leads to severe disability at an early age, while the juvenile form is more variable and its course is usually more protracted (Kehrer, 2011, Kehrer, 2014). MRI reveals name-giving leukodystrophic white matter changes in T2-weighted sequences, which can also be quantified visually or automatically for progression assessment (Groeschel, 2011). The associated metabolic changes in the brain make MR spectroscopy (MRS) a potential tool to monitor disease activity, relate clinical symptoms, as well as expand understanding of the pathophysiology. Especially regarding the evaluation of treatment approaches like haematopoietic stem cell transplantation, enzyme replacement or gene therapy for MLD (Krageloh-Mann and Groeschel, 2016, Fumagalli, 2022), objective assessment markers from the field of MR imaging are necessary.

MRS has been used in MLD patients since the 90 s (Kruse, 1993), yet was subject to limitations and lacked comparability due to the low incidence of the disease and technical challenges in MR sequences. With advancing MR hardware and software developments, its usefulness has been re-discovered in recent years, especially in the assessment of treatment effects. In a recent study, MR spectroscopy was shown to be able to retrospectively predict disease progression after stem-cell transplantation in patients with juvenile and adult onset (van Rappard, 2018, Krageloh-Mann, 2013). Creatine + phospohcreatine could distinguish poor from moderate and good outcome, the sum of glutamate and glutamine (Glx) could distinguish good from moderate and poor outcome, and *N*-acetylaspartate (NAA) could distinguish all outcome groups. The ratio of choline/*N*-acetylaspartate was shown to correlate in follow-up measurements in patients with moderate and good outcome (van Rappard, 2018). Further evidence of the potential clinical value of NAA was given by a good correlation to motor and cognitive functions in late-infantile patients using MRS in deep white matter (i Dali, 2010). Derived from these data, NAA has been implemented in some clinical trials for MLD as endpoint (C, i.d.,, 2020, C, i.d.,, 2021). Furthermore, NAA was found to be a biomarker for neurodegeneration in MLD that is increased in urine compared to controls (Laugwitz, 2022). However, it is not known whether the elevation of NAA in urine correlates with imaging data respectively.

Limitations of the current literature persist in small MLD patient cohorts with a confined assessment of the clinical relevance of MRS parameters across early- and late-onset patients. Furthermore, as mentioned above, the functional relevance of different regions in the brain has only partly been addressed for MLD. In addition to pathological changes, there are physiological variations as well as measurement conditions that can significantly influence the intra- as well as interindividual measurement variability of MR spectroscopy; for example, physiological variations of the aforementioned metabolite NAA have been reported with 5–20 % (Wellard, 2005).

The previous study data on MLD and MR spectroscopy were often collected with PRESS sequences, which have disadvantages in bandwidth and detection of metabolites with a short T2 relaxation time compared to modern sequences like LASER or semi-LASER sequences (Wilson, 2019).

Our purpose was to complement existing knowledge about MR spectroscopy in MLD by correlating clinical parameters for motor and cognitive performance in late-infantile as well as juvenile patients with MR spectroscopy data.

- in different regions of the brain to connect their neuroanatomic function with metabolic changes
- using a semi-LASER sequence at 3T and evaluating the spectra with a model-free, interval integration approach
- and to correlate these results with NAA concentrations in urine samples assessed with high-field (600 MHz) NMR spectroscopy.

## 2 Material and methods

### 2.1 Subjects

We used a semi-LASER MRS-sequence from 2013 to 2019 at the University Hospital Tübingen to collect data from patients. The diagnosis of MLD was confirmed by genetic testing and/or a deficiency of the ARSA enzyme with an increase in urinary sulfatide excretion, along with typical clinical features.

This study was approved by the local ethics committee (421/2021B01). Written consent was given by the subject or their legal guardian beforehand.

Study cohort: 29 MLD patients were included (median age 13.8 years, range 35 years, min. 2.1 years, max. 37.1 years), thereof 10 with late infantile (median age 3.8 years, range 3,7 years, min. 2.1 years, max. 5.9 years, 5 female) and 19 with juvenile (median age 15.9 years, range 29.9 years, min. 7.3 years, max. 37.1 years, 9 female) onset of the disease. 12 juvenile patients were treated with stem cell transplantation (median time between therapy and scan 1.6 years, range 12.1 years, min. −0.5 years, max. 11.6 years), the other patients were observed during the natural course of the disease. 11 juvenile patients were examined several times (2-6x, median time between scans 1.1 years, range 3.6 years, min. 0.4 years, max 4.0 years). All in all, 53 patient scans were analysed. As a control group, we included 12 healthy individuals (median age 18.5 years, range 49 years, min. age 10 years, max. age 59 years, 9 female) with normal motor and cognitive development (Groeschel, 2016).

To objectify motor function patients and controls were assessed via the gross motor function score in Metachromatic Leukodystrophy (GMFC-MLD-score (Kehrer, 2011) from 0 to 6 (0 = walking without support with quality of performance normal for age; 1 = walking without support but with reduced quality of performance; 2 = walking with support; 3 = Sitting without support and locomotion such as crawling or rolling; 4 = sitting without support but no locomotion or sitting without support not possible, but locomotion still possible; 5 = no locomotion nor sitting without support, head control is possible; 6 = no locomotion possible, loss of head control) which was documented for each 53 scanning time points. IQ was derived in juvenile patients from the Wechsler Intelligence Scale for Children 4th Edition (WISC-V) or Wechsler Adult Intelligence Scale 4th Edition (WAIS-IV). Absolute IQ values were available for 20 data sets, while for 40 scans it was possible to differentiate between an IQ of lower or higher than 85 (lower end of normal intelligence [100 – standard deviation] that is required for normal schooling as a prerequisite for the assumption of an IQ > 85; moreover for these patients there was no report of any cognitive problems by parents and physicians as defined before (Groeschel, 2016).

## 3 Procedures

### 3.1 MRI acquisition

Patients and controls were examined in a 3 Tesla scanner (Skyra or Prisma, Siemens Healthineers, Erlangen) at the University hospital in Tübingen with an extended sequence protocol consisting of a semi-adiabatic localisation by adiabatic selective refocusing (semi-LASER) CSI sequence (Groeschel, 2016) positioned above the lateral ventricles (TA = 3:57 min, TR/TE = 1600/135 ms, FoV = 160 × 160mm^2^, slice thickness = 12 mm, 12 × 12 acquisition matrix). After zero-filling of the raw data, a 32 × 32 matrix of spectra with 1024 data points each were obtained from a volume of interest of 160 × 160 × 15 mm^3^.

**Fig. 1:**
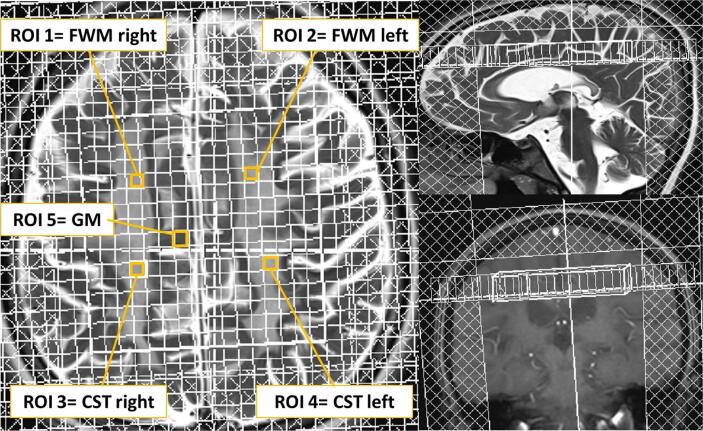
Shows positioning of the CSI grid above the lateral ventricles in axial (left), sagittal (right upper image) and coronar (right bottom image) view. On the axial slice the 5 evaluated regions of interest (ROI) are indicated. The mean value of ROI 1 and 2 was combined as frontal white matter (FWM), of ROI 3 and 4 as corticospinal tract (CST), of ROI 1–4 as white matter (WM) and ROI 5 is equivalent to grey matter.

For each patient, five voxels in defined anatomical areas (see Fig. 1) were selected using conventional T2- and T1-weighted images as described above (Groeschel, 2016) – two within the frontal white matter (FWM right and left), two were aligned with the right and left corticospinal tract (CST) in the supraventricular white matter with the posterior end under the sensorimotor cortex, one in the grey matter (GM) preferably of the parasagittal cortical zone in the right frontal hemisphere. The respective ROIs of both hemispheres were averaged, as MLD affects both hemispheres equally.

The corresponding free induction decay (FID) data were extracted from the CSI data set. An exponential filter (FWHM = 355 ms) was applied to the FID data. After Fourier transformation, the NAA peak was identified and the spectral distance of the NAA peak from the expected position of 2 ppm was evaluated. A frequency correction of the spectra was performed by shifting the spectra by this spectral distance. A phase correction was not necessary. A baseline offset was determined for each spectrum as the mean value in the spectral range between 0.9 and 1.6 ppm and subtracted from the spectra. After this postprocessing, the spectra were averaged for the examined patient and control groups.

**Fig. 2:**
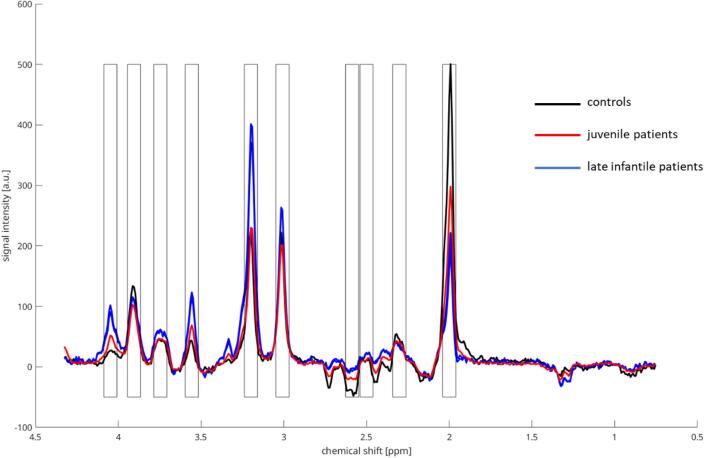
Shows an exemplarily overlay of averaged spectra (roi 3) for controls (black), juvenile (red) and late infantile (blue) patients to visualize the localisation of the selected 10 frequencyintervals of interest (IOI) which are oriented towards 10 main peaks of the spectrum. Patients differ from controls with higher or lower signal intensities in most of the intervals. (For interpretation of the references to colour in this figure legend, the reader is referred to the web version of this article.)

We then defined 10 intervals of interest (IOI) with a frequency range of each 0.08 ppm that were oriented towards 10 main peaks of the spectra (see Table 1 and Fig. 2). These peaks were related to single metabolite spectra (Govindaraju et al., 2000) and named after the metabolite primarily contributing. The absolute integral of each interval was further calculated and divided by the interval related to the main Creatine peak (Cr2), which was stable throughout the data sample and did not differ significantly between the 3 groups (controls, late infantile patients, juvenile patients). These ratios were used for the following analyses.

**Table 1:** Shows the 10 intervals that were defined along the MR spectra. Each interval was assigned to a main peak in the spectra with a spectral chemical shift of 0.08 ppm and named after the single metabolite mainly contributing to the respective peak.

Interval No.	Associated main peak	Abbreviation	Chemical shift [ppm]
1	Myo-Inositol 1	Myo1	4.087–4.008
2	Creatine 1	Cr1	3.944–3.865
3	Glutamate/Glutamine	Glx	3.786–3.706
4	Myo-Inositol 2	Myo2	3.595–3.516
5	Choline	Cho	3.238–3.159
6	Creatine 2	Cr2	3.048–2.968
7	Aspartate	Asp	2.627–2.548
8	N-Acetylaspartate 1	NAA1	2.540–2.460
9	Glutamine	Gln	2.341–2.262
10	N-Acetylaspartate 1	NAA2	2.040–1.960

To compare performance and variance with the gold standard of a single metabolite spectrum, the area CST left was evaluated and compared for all controls using both the conventional method and the evaluation used in this study. For this purpose, the coefficient of variance (mean/standard deviation) was calculated for metabolites derived from the LCModel and their related interval of interest (Ins = Myo2; GPC + PCh = Cho; aspartate = Asp; glutamine = Gln; NAA = NAA2).

Bland Altmann plots comparing the above mentioned pairs of metabolite and IOI showed that 98.3 % of data points lie within the limits of agreement (mean difference ± 1.96 × standard deviation of the differences). Therefore, a comparability of the two methods can be assumed. Spectrum analysis using the LCModel failed to delineate a signal for aspartate in 11 of 12 data sets and in 2 of 12 for glutamine. Coefficients of variance were comparably low between both measurements (Asp 0.2 vs aspartate 3.31 [as mentioned above many data points missing], Cho 0.1 vs GPC + PC 0.07, Myo2 0.19 vs Ins 0.2, Gln 0.13 vs glutamine 0.79, NAA2 0.1 vs NAA 0.21. Although only comparable to a limited extent, NAA2 correlated significantly with NAA of the LCModel (r = 0.61, p = 0.035) and choline with GPC + PCh of the LCModel (r = 0.74, p = 0.006). Aspartate, myo-inositol and glutamine did not correlate significantly with each other.

For the remaining data, only the interval integration approach was applied. The analysis was performed on an individual patient level and group levels (healthy controls, patients with MLD of the late-infantile form, and those with the juvenile form). As we hypothesize a symmetrical white matter involvement in MLD, we compared a mean value of all 4 white matter regions between patients and controls. For correlation with motor function, a mean value of both CST regions was used, and for correlation with cognitive function, a mean value for the FWM regions, as we hypothesize a stronger structure function relationship in these regions, as done with T2-hyperintensities in MLD before (Strolin, 2017).

ANOVA tests were performed with post-hoc analysis using Tukey test for differences between groups. Correlation of MRS with clinical parameters was performed using Spearman's rank correlation coefficient (r_s_) for the categorical parameter GMFC-MLD and Pearson's correlation coefficient (r) for the continuous variable IQ. For all tests, the significance level was defined as p < 0.05. Values for GMFC-MLD at the time of scan were available for all MR-datasets.

## 4 Nuclear magnetic resonance (NMR) spectroscopy of urine samples

The detailed analysis is described elsewhere in detail (Laugwitz, 2022). In summary, ^1^H NMR spectra were acquired via the Bruker Avance IVDr 600 MHz system (Bruker Avance III HD, Ettlingen, Germany) and analysed using Bruker’s B.I.Quant-UR1.1 module for quantification of urine metabolites using the MetaboAnalyst 5.0 Toolbox dedicated for raw data processing (Pang, 2021). NAA and Creatinine were quantified without further normalization as raw values in mmol/l volume of interest and set in relation to each other. Corresponding urinary NAA concentrations measured by NMR were available for a subset of 21 MR datasets. The time interval between the urine sampling date and MR spectroscopy was 0.8 years on average with a standard deviation of 1.2 years.

## 5 Results

### 5.1 Group comparison

**Fig. 3:**
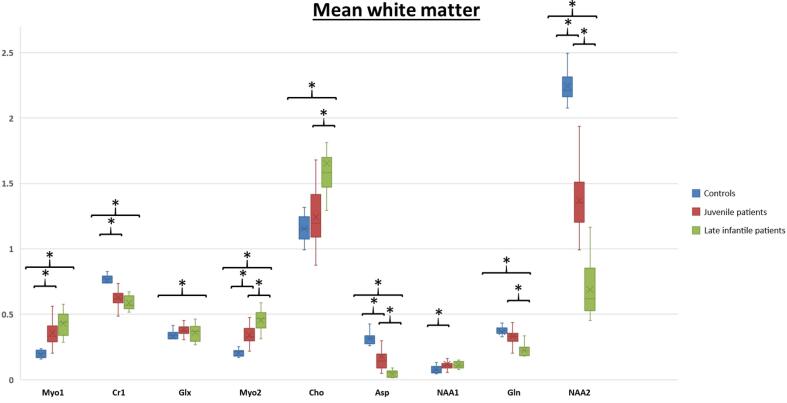
Box plots of controls, juvenile, and late-infantile patients are shown for 9 IOIs of the MRS spectra derived from white matter (mean of FWM and CST). Asterisks above curly bracket indicate a significant difference between the area under the curve (AUC) of controls, juvenile and late infantile patients.

Fig. 3 shows the statistical group comparisons, comparing controls with juvenile patients and juvenile patients with late-infantile patients, respectively. Myo1, Myo2, and Cho are elevated in patients, while the other parameters are decreased, which is most pronounced in NAA2. Glx and NAA1 are the IOIs with the least significant results. Grey matter ROI (not shown here) only shows few significant IOIs. For all other IOIs, late infantile patients differ significantly from the control cohort. In general, the differences to the control group are accentuated in late infantile patients. Juvenile patients show a similar pattern with a few less significant IOIs (Cho and Gln in CST and WM, respectively). Late infantile patients differ significantly from juvenile patients for Myo2, Cho, Asp, Gln and NAA2 (for details see Table 2).

**Table 2:** Statistical results for ANOVA tests with post-hoc analysis using Tukey test for differences between groups controls vs juvenile patients, controls vs late infantile patients and juvenile vs late infantile patients, for each interval of interest (IOI) white (WM) and grey matter (GM). Significant p-values are highlighted in green. Moreover, correlation between GMFC-MLD in CST and IQ, group of IQ higher or lower than 85 (IQ 85) in FWM for each IOI is shown. Significant correlations are each highlighted in colours (GMFC-MLD yellow, IQ light blue, IQ 85 brown).

In the control group there was no significant correlation between age at scan and any of the IOIs (see supplementary Fig. 1).

### 5.2 Clinical parameters

**Fig. 4:**
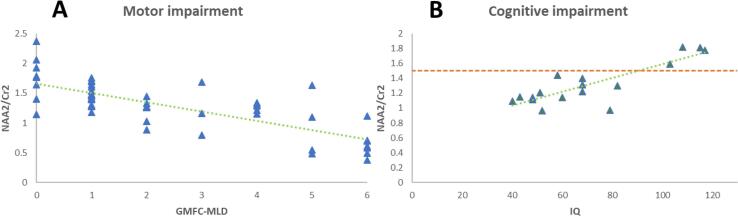
A – The left diagram shows the negative correlation (r_s_ = −0.75; p < 0.001) of NAA2 in the mean CST to the GMFC-MLD scores. Higher motor impairment went with decreased values for NAA2. B – On the right side, NAA2 is set in correlation with absolute IQ values. Lower IQ as expression of cognitive impairment showed low values for NAA2 (r = 0.84, p < 0.001). A value of 1.5 (green line) differentiated patients with an IQ < 85 and those with an IQ > 85 with and odds ratio of 18 in the mean frontal white matter. (For interpretation of the references to colour in this figure legend, the reader is referred to the web version of this article.)

The GMFC-MLD score correlated significantly with many IOIs of the spectrum. The strongest correlation is for NAA2 in the CST. The mean values from both hemispheres correlated negatively with GMFC-MLD values (r_s_ = −0.75; p < 0.001). Fig. 4A visualizes this negative correlation of decreasing NAA2 values and increasing GMFC-MLD and thus increasing motor impairment.

The absolute IQ value, on the other hand, correlated likewise significantly with NAA2 (r = 0.84, p < 0.001) in the mean frontal white matter, so that a higher value for the NAA2 spectral area was observed at higher IQ (see Fig. 3B).

Differentiating the patients into two groups of IQ > 85 and IQ < 85, the NAA2 IOI again showed the best correlation. In mean, frontal correlation is highest with r_s_ = 0.55 (p < 0.001). Thus, again a higher NAA value indicated a higher IQ value. NAA2 of 1.5 discriminated with an odds ratio of 25 between an IQ < 85 and an IQ > 85.

**Fig. 5:**
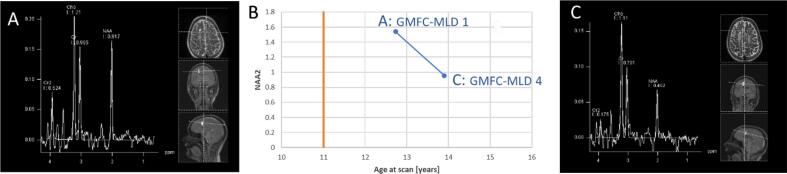
Longitudinal follow-up measurement of a patient with natural course of disease and juvenile onset (marked in B with an orange line at about 11 years) who experienced rapid deterioration in cognitive and motor function from the second year after disease onset, deteriorating from a cognitive level with still normal schooling (A) to speaking only two-word phrases (C) and a motor decline with a GMFC-MLD of 1 to 4 within a further year. A and C compare the raw spectra from ROI 1 (right frontal white matter). The NAA peak from A to C is clearly reduced, even in relation to the creatinine peak, correlating with the cognitive and motor deterioration. (For interpretation of the references to colour in this figure legend, the reader is referred to the web version of this article.)

Follow-up measurements did not show a common trend of the metabolites over time as time points of measurements were totally unrelated to date of onset of the disease or clinical symptoms. Moreover most patients remained clinically relatively stable (most of these patients were treated with stem cell transplantation). Fig. 5 shows an example of a longitudinal follow-up of one patient with a natural course of the disease who clinically deteriorated between two measurements both in motor function and cognitive function, illustrating the longitudinal changes in the MR spectrum.

### 5.3 Comparison to urine NMR spectroscopy

Based on the above-shown results, NAA was chosen as the most informative parameter for comparison. Urinary NAA concentrations were evaluated as a ratio to Creatinine and correlated with the value for NAA2 from MRS.

**Fig. 6:**
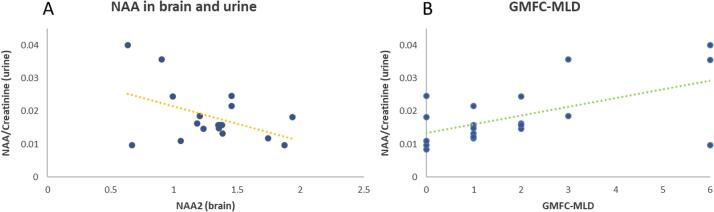
A: NAA2 in the mean white matter is negatively correlated with NAA levels in urine quantified as ratio of NAA/Creatinine. Decreased levels of NAA in the brain go along with a higher excretion of NAA in urine. B: Urine NAA/Creatinine is significantly correlated with the GMFC-MLD. Lower levels of NAA/Creatinine indicate a higher degree of motor impairment.

There was a significant negative correlation everywhere except for the grey matter with a maximum value of r_s_ = −0.55 (p = 0.01) in the mean white matter. A lower value for NAA2 is therefore associated with higher NAA levels in the urine (see Fig. 6A).

Moreover, urine NAA/Creatinine correlated significantly (r_s_ = 0.46; p = 0.038) with the motor impairment measured as GMFC-MLD (see Fig. 6B). There was no significant correlation with the IQ.

## 6 Discussion

In this study, we could show that the presented model-free approach of analysing MR spectroscopy data, derived from a semi-LASER sequence at 3T, can be used to differentiate between healthy controls and our MLD cohort of more (late infantile) and less severely affected (juvenile) patients. At the same time, MR spectroscopy is suitable to reflect clinical parameters of motor and cognitive symptomatology during the course of the disease. NAA was demonstrated to be the most significant parameter showing good correlations with GMFC-MLD and IQ of r_s_ = −0.75 and r = 0.84, respectively. In addition, this NAA-IOI correlated negatively with the urinary NAA/creatinine concentration of the patients as determined by urine MR spectroscopy. Compared to the analysis of single metabolites in the LCModel, the interval integration-based analysis shows more extractable results, slightly lower coefficients of variance paired with good clinical results and therefore appears suitable as an alternative evaluation method for individual or group comparisons yet not for single metabolite analysis.

The IOI of Glx and NAA1 turned out to be least different from the control group, while all other IOIs yielded strongly significant differences. Next to NAA2, Myo1, Cr1, Myo2, Cho, Asp and Gln were well suitable to differentiate patients from controls. Late infantile patients could significantly be delineated from juvenile patients via Myo2, Cho, Asp, Gln and NAA2.

When investigating the correlation with clinical symptoms, several IOIs succeeded in correlating strongly to motor symptoms, including in particular for differentiating all 3 groups in CST Myo1, Myo2, Asp and NAA2. NAA2 achieved the best correlation with r_s_ = −0.75. With increasing motor deficits, NAA2 decreased accordingly. Cognitive symptoms correlated to a lesser extent than motor symptoms with brain metabolites. As expected, the best correlation was found in the frontal white matter and NAA2 with r_s_ = 0.84 (p < 0.001) (and to a lesser extent for Asp and Gln). Comparing patients with normal IQ to patients with an IQ below 85, lower NAA values were clearly associated with worse cognitive function with an odds ratio of 25 for a value of NAA2 = 1.5.

Interestingly, the post-hoc analysis of NAA2 in the brain of MLD patients revealed a good correlation (r_s_ = −0.55, p = 0.01) with elevated NAA/creatinine excretion in urine. Moreover the ratio of NAA/Creatinine in urine correlated significantly with the GMFC-MLD as a measure of motor impairment. These results are in line with a recent study evaluating NMR urine profiling in metachromatic leukodystrophy (Laugwitz, 2022). Here, NAA in urine was elevated in MLD patients in comparison to controls, especially in the cohort with early onset MLD who exhibited the most progressive neurodegenerative disease course. In a few single case follow-ups, it was shown that patients with juvenile MLD who stabilize clinically after HSCT, reveal a decrease or even normalization of NAA in urine. Furthermore, patients with a disease progression after HSCT exhibit increased NAA levels in urine. We hypothesize that high NAA is an indicator of healthy brain and that NAA is only excreted when there is neuronal damage. This would at least explain the inverse correlation between NAA in brain and urine. Nutritional supplementation of NAA was excluded. As the peripheral nervous system can be affected in MLD as well (Gieselmann and Krageloh-Mann, 2010), elevated NAA excretion in urine might in parts be supported by a peripheral neuropathy. NAA has been described as a relatively exclusive neuronal metabolite with only marginal concentrations in peripheral tissues (Bogner-Strauss, 2017), so it seems unlikely that other organ manifestations of MLD like gastrointestinal disorders would influence the NAA excretion in urine in a relevant way. Yet it is possible that the ratio of NAA/creatinine could be influenced by a change of creatinine over time, since the excretion of creatinine in urine is dependend on muscle activity and volume. In a bedridden stage of disease it is possible that creatinine in urine decreases due to inactivity and muscle atrophy which could elevate the ratio of NAA/creatinine. Yet the so far observed values for MLD patients are about 6 times higher than in a normal control group comprising 323 probands (Laugwitz, 2022) so a theoretic effect of creatinine that can also only be assumed in end stages of the disease should be marginal compared to the amount of pathological elevation of NAA. An elevation of NAA in urine has also been observed for Canavan disease as another genetic leukodystrophy (Al-Dirbashi, 2007).

NAA is synthesized from aspartate and acetyl coenzyme A (acetyl CoA) in neurons, as it serves as a source of acetate for lipid and myelin synthesis in oligodendrocytes and is a precursor of the neurotransmitter *N*-acetylaspartylglutamate. NAA is therefore considered as a correlate for neuronal integrity (Moffett, 2007, Oz, 2014). Yet NAA has also been found in oligodendrocytes, myelin, and axons (Nordengen, 2015). The fact that the greatest difference in NAA concentration was detectable in white rather than grey matter, argues for NAA being a clinically meaningful parameter for MLD white matter damage. Whether this functional loss due to NAA reduction argues for NAA being a neuronal (axonal) or oligodendrocyte damage can only be speculated about, probably both cell types are affected as they work as a functional unit (Groeschel, 2012, Piaton et al., 2010). It is conceivable that NAA/creatinine in urine quantified via MR spectroscopy or tandem mass spectrometry (Al-Dirbashi, 2007) might be useful as a biomarker for neurodegeneration with regard to prospective information about the course of the disease in MLD. However, this needs to be further investigated in more extensive studies.

In a literature review of the existing MRS studies in MLD, the ability of MRS to correlate to motor and cognitive symptoms can be reproduced in other cohorts. I Dali et al showed in late-infantile patients a correlation of NAA to the GMFM score of 0,89 in the centrum semiovale and of NAA to Mullen scales of early learning of 0,81 in the parieto-occipital white matter (i Dali C. 2010), which is in comparable dimensions to our results for the total cohort. On the other hand, Rappard et al (van Rappard, 2018) focused in their analysis on the outcome after stem-cell transplantation, yet showed a correlation of GMFC-MLD to NAA of −0,78 in white matter. NAA was also shown to be a good differentiating marker between good/ moderate/ poor outcome, whereas this was still partially possible for creatine and the sum of glutamate and glutamine (Glx). Myo-inositol and choline were reported as elevated (van Rappard, 2018, Assadi, 2013) in line with the present results. Lactate as an additional metabolite was found to be elevated in MLD patients (Kruse, 1993, Assadi, 2013). In our approach, a small negative signal in the lactate region is visible in the average spectra, but in most of single spectra, the lactate signal could not be separated from the noise background. Therefore, the evaluation of the lactate region was not included in this evaluation. Compared to established MR parameter for clinical correlations, MR spectroscopy achieves similar results to MLD sum score or demyelination load (Strolin, 2017, Groeschel, 2012, Tillema, 2015). Along these lines, it was shown before that demyelination load in the central region of the white matter, including the CST, correlated with motor function, and in the frontal white matter, with cognitive function in patients with MLD (Strolin, 2017). On the other hand, it was shown that MR spectroscopy of NAA is not affected by the phenomenon of T2 pseudo-normalization in the late course of the late-infantile form of disease, emphasizing the added value of quantitative microstructural MR parameter for MLD (Martin, 2020). However, it should be noted again that the IOIs measured by the current method include portions of other metabolites and therefore do not include only the metabolite representing the particular peak. Thus, the results can only be applied with restrictions to other studies where the results are based on single spectra according to the LCModel.

The applied model and fitting free interval integration approach to analyse MRS data suited well to detect and quantify pathological changes in MLD patients throughout the different courses of the disease and correlated well with clinical symptoms while showing smaller dimensions of variation compared to the more sophisticated single metabolite analysis using LCModel. Interval or peak integration is usually applied to spectra with sparse, non-overlapping peaks and less complex baselines – a circumstance that is not given in in-vivo ^1^H-MRS of the human brain, as it is not possible to differentiate the contribution of other metabolites to the integral of the frequency range (Near, 2021). Yet, as can be observed in the controls, some areas of the spectrum are not usable due to low separability of individual metabolites such as aspartate (11 of 12 controls with value of 0), whereas in the current approach the sum of the proportions of several metabolites in the respective integral is large enough to use it for clinical correlations. Thus, for this very aspartate, which could virtually not be delineated with LCModel, one of the most relevant IOIs besides NAA2 could be established with the current approach. However, only a limited statement can be made about individual metabolites – in the case of the NAA peak, which is very dominant in the spectrum, this is perhaps still possible, which is also shown by the good correlation with NAA in the LCModel. In the case of other metabolites – especially those with mulitple and low peaks such as myo-inositol – this is no longer possible to the same extent. The approach presented here would therefore in principle be well suited to use MR spectra to distinguish between groups or individuals using multiple sections of the spectrum, but without actually going down to the level of individual metabolites.

Advances in MR scanner hardware and software have allowed the implementation of robust MRS sequences. In contrast to previous MRS studies in MLD, a semi-LASER sequence was used in the current protocol. Compared to other MRS sequences such as PRESS or STEAM, this is characterised by a lower chemical shift displacement error, which enables better localisation in the context of single voxel spectroscopy. In addition, the semi-LASER sequence enables better detection of metabolites with complex mulitplets such as glutamate (Wilson, 2019). It is available for scanners from Siemens, Philips and GE. There might be minor differences in the timing of the used radiofrequency pulses, but this should not alter the effects described in this study. We can show that the application of a semi-LASER sequence is feasible in a clinical context and yields reliable results in MLD patients which could be transferred to other Leukodystrophies.

This study offers clinical implications: The presented interval of interest-based spectrum analysis is suitable to derive clinically valuable information. Whether this actually succeeds to a better extent than with single metabolite analyses needs to be shown by supplementary studies. In any case, especially NAA2 in CST and FWM is suitable as a meaningful parameter to reflect the clinical symptoms of MLD patients. Interestingly, there is an intermediate correlation to NAA concentration in urine and between NAA in urine and motor impairment, so this could also reflect disease activity in the brain as an even less burdensome measurement method for the patient. This is particularly interesting for monitoring approved or experimental therapeutic approaches and a potential easy to access biomarker. In regard to the therapies that are fortunately available it seems reasonable and necessary to quantify disease activity and a possible therapy response (or failure) in a more differentiated way than with the 7-step GMFC-MLD scale or IQ tests. The recently described parameters neurofilament light chain (Nfl) and glial fibrillary acidic protein (GFAP) in CSF and blood (Beerepoot, 2022) or – as described here – NAA in urine are potential biomarkers for this purpose. It would be potentially important to better investigate these parameters for the use in new born screening or prediction of phenotypes. In addition, MR-based evaluation has the advantage of correlating clinical symptoms directly with the anatomical region rather than systemically (e.g., cognitive function with the FWM, motor function with the CST). For this purpose, the MR-based quantification of T2 hyperintensities is available, which, however, as described before (Martin, 2020), can also be decoupled from the course of the disease. MRS and diffusion-based evaluation were proven to show pathological changes in still normal appearing white matter (van Rappard, 2018, van Rappard, 2018) so that much more subtle changes can be detected by both of these methods probably having the potential to anticipate the clinical course to come to a certain extent and therefore help to guide therapeutic decisions.

This study does not yet provide insights into the metabolic course under therapy. Although several patients have been treated with stem cell transplantation, the focus of the study is on the correlation of MRS with clinical parameters and not on the response to therapy. It cannot be excluded that a patient population with a purely natural course of the disease will show more pronounced changes. However, it is very difficult to form a homogeneous group in sufficient numbers due to the rarity of the disease. This resulted also in the inclusion of follow-up scans in the analysis which might potentially influence the cross-sectional statistical analysis. We provide in the supplements the evaluation for the independent data (for patients with follow-up measurements, only the most recent data set was included) for comparison (supplementary Fig. 2 and supplementary Table 2). A similar pattern emerges in the group comparison and in the correlation for the GMFC-MLD with the total cohort, while the statistical power is clearly lower (for IQ only 8 instead of 20 data points and for urine MR spectroscopy only 11 instead of 21 data points left) and thus not all correlations remain significant. However, the results yielded with the total cohort were in line with the so far published literature about MR spectroscopy of brain and urine in MLD. In order to analyse larger patient cohorts, multi-site analyses would be necessary and desirable.

Another weakness of the study is that the control cohort is of a similar age range, but not exactly age-matched. The age effect in the control cohort was investigated and showed no significant correlation between age at scan and any of the intervals of interest (supplementary Fig. 1). Given the high effect size of the differences between patients and controls, it seems that the age related effect is minor. However, especially for the late infantile group, it cannot be excluded that age effects, in addition to the effects of the pathological changes, influence the difference to the control and juvenile cohort.

### 6.1 Conclusion

This study adds to our current understanding on MRS in MLD

- the neuroanatomical connection of metabolic changes in different regions of the brain and their respective correlation to clinical symptoms.
- the applicability of a semi-LASER sequence in MLD and an alternative model-free post-processing approach that is well suited to differentiate between the different cohorts.
- the correlation between changes of NAA in the brain and in urine, which recommends the latter one to further studies about its potential as biomarker for MLD and other neurodegenerative diseases.

## Funding

This work was supported by DFG grant GR 4688/2–1 and by an investigator-initiated research grant from Takeda Pharma AG (IIR-DEU-002540). We acknowledge support by Open Access Publishing Fund of University of Tübingen. In addition, we wish to thank all participating German Leukonet centers. S.G. and I.K.M. are members of the European Reference Network for Rare Neurological Diseases – Project ID No 739510.

## 8 Ethics approval

The study was approved by the Ethical Committees of the University of Tuebingen, Germany. Written informed consent was given by the parents of the patients or the patients themselves.

## CRediT authorship contribution statement

**Joana Feldmann:** Investigation, Formal analysis, Writing – original draft. **Pascal Martin:** Investigation, Formal analysis, Writing – original draft, Writing – review & editing, Visualization. **Benjamin Bender:** Investigation, Validation, Writing – review & editing. **Lucia Laugwitz:** Investigation, Writing – original draft, Writing – review & editing. **Laimdota Zizmare:** Investigation, Validation. **Christoph Trautwein:** Writing – original draft, Writing – review & editing. **Ingeborg Krägeloh-Mann:** Resources, Conceptualization. **Uwe Klose:** Conceptualization, Methodology, Software, Writing – review & editing, Visualization. **Samuel Groeschel:** Resources, Conceptualization, Methodology, Writing – review & editing, Supervision, Project administration, Funding acquisition.

## Declaration of Competing Interest

The authors declare the following financial interests/personal relationships which may be considered as potential competing interests: S.G. received institutional research support from Shire international GmbH, outside of the submitted work. He is an advisor and co-investigator for trials in Metachromatic Leukodystrophy (Shire international GmbH, Orchard, Bioclinica), but receives no personal payment related to this role. B.B. is a co-founder and shareholder of AIRAmed GmbH, with activities outside of the submitted work. P.M. has received honorary as an advisory board member from Biogen unrelated to the submitted work. The other authors declare that they have no conflict of interest.

## Data Availability

Data will be made available on request.
