# Supplementary material for: MR-spectroscopy in metachromatic leukodystrophy: A model free approach and clinical correlation
Source: Neuroimage Clin. 2022 Dec 20;37:103296. doi: 10.1016/j.nicl.2022.103296 (PMC9800432; doi:10.1016/j.nicl.2022.103296)
Supplement: Supplementary data 1 [file mmc1.docx]

# **Supplements**


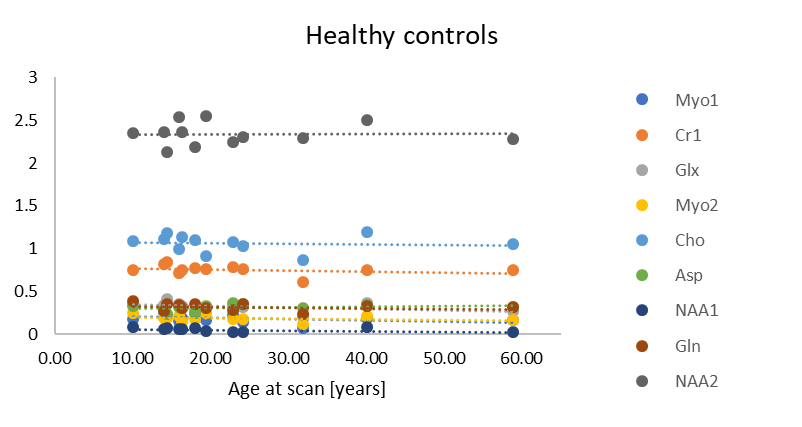


*Supplementary figure 1: There is no significant correlation between age at scan and any of the intervals of interest for controls in any ROI, here shown for mean white matter.*


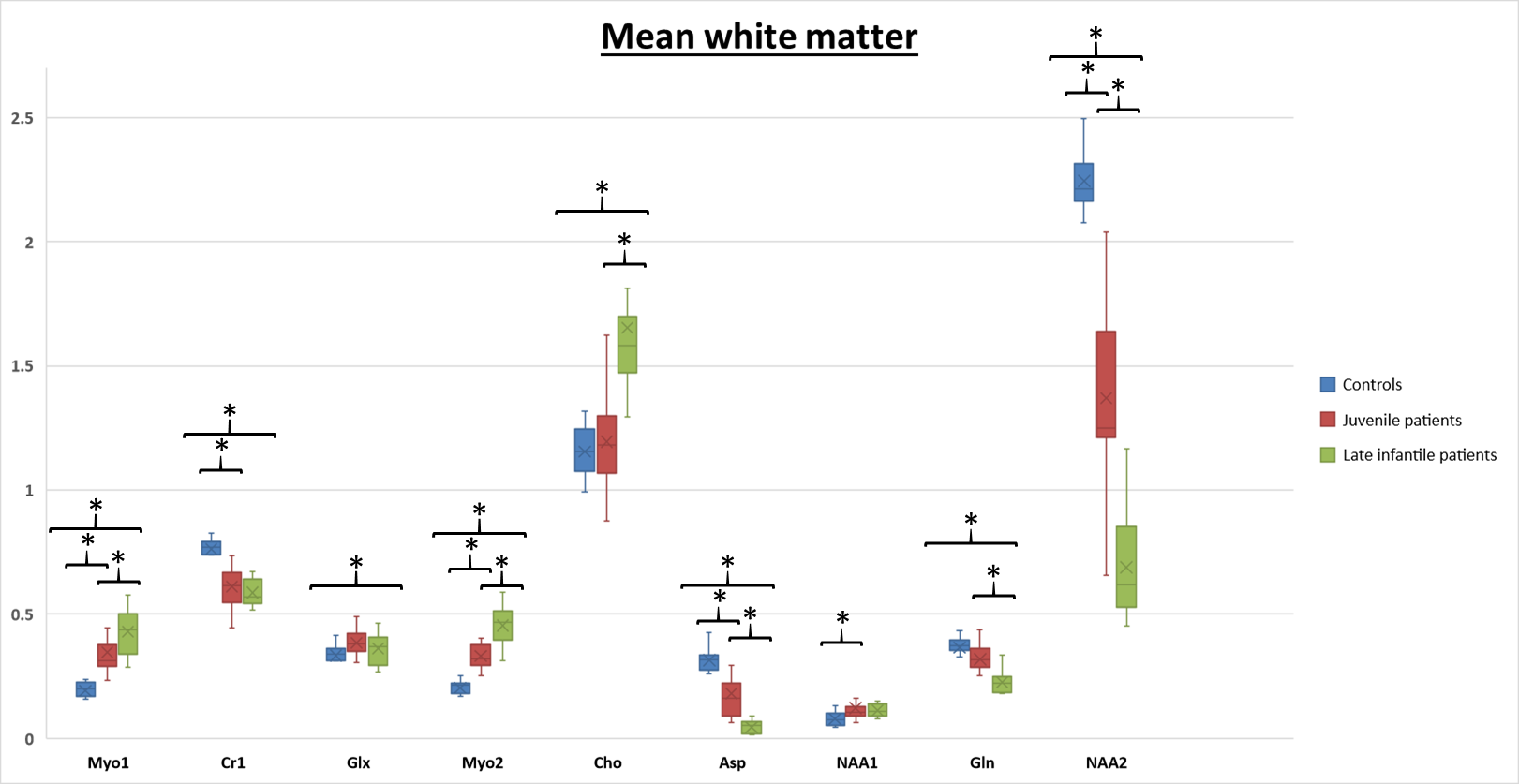


*Supplementary figure 2: Box plots of controls, juvenile, and late-infantile patients are shown for 9 IOIs of the MRS spectra derived from white matter (mean of FWM and CST). Asterisks above curly bracket indicate a significant difference between the area under the curve (AUC) of controls, juvenile and late infantile patients. In contrast to figure 3, analysis for these results included only independent scans from patients (10 late infantile patients, 19 juvenile patients).*


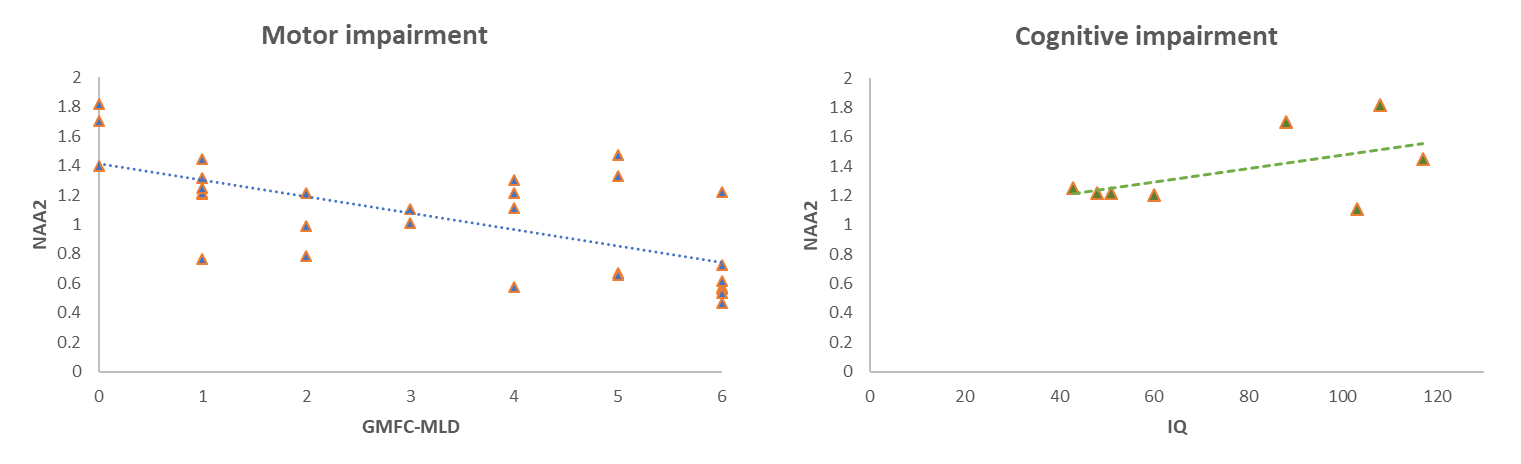


*Supplementary figure 3: In contrast to figure 4, analysis for these results included only independent scans from patients (10 late infantile patients, 19 juvenile patients). The left diagram (A) shows the negative correlation (r*_s_*= -0.83 p<0.001) of NAA2 in the mean CST to the GMFC-MLD scores. Higher motor impairment went along with decreased values for NAA2. B - On the right side, NAA2 is set in correlation with absolute IQ values. Lower IQ as expression of cognitive impairment showed a trend towards low values for NAA2 (r = 0.53), yet did not reach statistical significance anymore (p= 0.174) compared to the total group with 20 data sets.*

Supplementary table 1: *Statistical results for ANOVA tests with post-hoc analysis using Tukey test for differences between groups controls vs. juvenile patients, controls vs. late infantile patients and juvenile vs. late infantile patients, for each interval of interest (IOI) in white (WM) matter and grey matter (GM). In contrast to table 2, analysis for these results included only independent scans from patients (10 late infantile patients, 19 juvenile patients). Significant p-values are highlighted in green. Moreover, correlation between GMFC-MLD, IQ, group of IQ higher or lower than 85 (IQ 85) and each IOI is shown for CST and FWM. Significant correlations are each highlighted in colours (GMFC-MLD yellow, IQ light blue, IQ 85 brown).*
